# Stimulus novelty, task demands, and strategy use in episodic memory

**DOI:** 10.1177/1747021820980301

**Published:** 2021-01-09

**Authors:** Otto Waris, Daniel Fellman, Jussi Jylkkä, Matti Laine

**Affiliations:** 1 Department of Psychology, Åbo Akademi University, Turku, Finland; 2 Department of Child Psychiatry, University of Turku and Turku University Hospital, Turku, Finland; 3 INVEST Research Flagship Center, University of Turku, Turku, Finland; 4 Department of Applied Educational Science, Umeå University, Umeå, Sweden; 5 Department of Clinical Neuroscience, Karolinska Institute, Stockholm, Sweden; 6 Turku Brain and Mind Center, University of Turku, Turku, Finland

**Keywords:** Memory strategy, episodic memory, list learning, mnemonics, task novelty, task difficulty

## Abstract

Cognitive task performance is a dynamic process that evolves over time, starting from the first encounters with a task. An important aspect of these task dynamics is the employment of strategies to support successful performance and task acquisition. Focusing on episodic memory performance, we: (1) tested two hypotheses on the effects of novelty and task difficulty on strategy use, (2) replicated our previous results regarding strategy use in a novel memory task, and (3) evaluated whether repeated open-ended strategy queries affect task performance and/or strategy use. The present pre-registered online study comprised 161 adult participants who were recruited through the Prolific crowdsourcing platform. We employed two separate 5-block list learning tasks, one with 10 pseudowords and the other with 18 common nouns, and collected recall performance and strategy reports for each block. Using Bayesian linear mixed effects models, the present findings (1) provide some support for the hypothesis that task-initial strategy development is not triggered only by task novelty, but can appear also in a familiar, moderately demanding task; (2) replicate earlier findings from an adaptive working memory task indicating strategy use from the beginning of a task, associations between strategy use and objective task performance, and only modest agreement between open-ended versus list-based strategy reports; and (3) indicate that repeated open-ended strategy reports do not affect objective recall. We conclude that strategy use is an important aspect of memory performance right from the start of a task, and it undergoes development at the initial stages depending on task characteristics. In a larger perspective, the present results concur with the views of skill learning and adaptivity in cognitive task performance.

## Introduction

It has been argued that cognition is a dynamic system that changes not only in the long run but also while in the midst of task processing (Beer, 2000; Riley & Holden, 2012; Smith & Thelen, 2003; Van Gelder, 1998). To be taken seriously, this would call for a paradigm shift in cognitive assessment, moving from the study of cognitive abilities as stable, invariable capabilities measured by summative scores, towards an inquiry of the cognitive dynamics that unfold during task performance. For example, when performing a memory task, individual skills and strategies interact with key task features such as novelty, processing requirements, and difficulty, and together these factors shape how the task is processed. Models of cognitive skill learning have separated three major phases in this process (Chein & Schneider, 2012). Upon encountering a novel task, the cognitive system enters the Formation stage, where the metacognitive system establishes strategies and behavioural routines that manage task performance. These processes are put into use in the Controlled Execution phase, which relies on the cognitive control system. Finally, task performance becomes gradually more automatic and modular in the Automatic Execution phase, and the resources of the Metacognitive and Cognitive Control systems are freed for other tasks (for similar accounts, see Baars, 1988, 2002; Dehaene & Changeux, 2011). With this general approach as our starting point, we studied the evolvement of strategies and objective task performance during two episodic memory tasks that varied in stimulus novelty, enabling us to examine how this factor affects the dynamics of episodic memory performance. In this context, we consider a strategy to be a thinking technique or a consciously chosen way of processing information that aids in the encoding and recall of the to-be-remembered information.

Concerning the skill learning view, Gathercole and colleagues (2019) recently proposed a so-called cognitive routine framework that takes a stance on the effects of novelty in memory task performance. Focusing on working memory and its malleability, Gathercole et al. (2019) argued that performing a novel task will lead to the development of new cognitive routines or strategies that control the sequence of cognitive processes required to perform the task. On the contrary, no new routines or strategies will be developed for familiar tasks, as their familiarity entails that they can be managed by pre-existing routines (the only exception would be fine-tuning of those routines following lengthy practice).

In support of this framework, Waris et al. (2021) recently showed that the use of strategies and the level of detail in the reported strategies increased during a working memory task (n-back) that was novel to the participants. However, that study lacked a control condition, that is, a familiar task that according to the cognitive routine framework should not generate these kinds of strategy developments. In the present pre-registered experimental study (see https://osf.io/24tcy for pre-registration), we employed two list learning tasks that varied in stimulus novelty, which allowed us to systematically test for possible differences in strategy use between these two conditions. The tasks were divided into five separate blocks, and by querying for strategy use after each block, we could examine the temporal pattern of strategy use across the task blocks, as well as the relationships between strategy use and objective recall. The novel task condition required the participants to learn a list of meaningless pseudowords, a task that would be very rarely practised in everyday life. The familiar task condition called for learning a list of common nouns, a well-known task in its various forms for most individuals (shopping lists, to-do lists, to-be-remembered instructions, to-be-learned lists such as names of people, places, living and non-living things, etc.).

Using the novel versus familiar list learning conditions, we pitted two hypotheses against each other. The first hypothesis was derived from the cognitive routine framework (Gathercole et al., 2019) that postulates that only a novel task leads to the development of new cognitive routines (i.e., strategies) that control the sequence of cognitive processes required to perform the task. In this study, this hypothesis predicts strategy development over the task blocks only in the pseudoword list learning condition (novelty hypothesis). More specifically, strategy development should be observed as increased and better detailed strategy use over the task blocks when trying to learn the pseudowords. Moreover, objective recall performance across the blocks should correlate positively with strategy use and strategy level of detail. In contrast, right at the start of the familiar word list condition, frequency of strategy use, level of strategy detail, and objective recall performance would be at a higher level than in the pseudoword condition, and the two strategy variables would not show any significant change over the blocks as the cognitive routines for this familiar task have been available already at the beginning. Note that Gathercole et al. (2019) hypothesise that for a familiar task like verbal serial recall, adoption of new strategies would only take place “under conditions of extensive and prolonged practice” (p. 23), which is clearly not the case with the present single-session study.

The second, competing hypothesis was that not only novelty but also task demands contribute to the development of novel routines and strategies (task demand hypothesis). Thus, even a highly familiar task can trigger strategy development when it is demanding enough so that the readily available cognitive routines or strategies do not suffice for managing it. This echoes in part an earlier proposal by Belmont and Mitchell (1987), according to which cognitive tasks perceived as moderately difficult (and not as easy or exceedingly difficult) are more likely to elicit strategic behavior. Accordingly, Hypothesis 2 predicts that even our word-list learning condition, including 18 to-be-learned items that clearly exceeds normal span limits, will show strategy development across the task blocks through increased frequency of strategy use and more detailed strategies. Note that the critical difference between the two hypotheses concerns strategy use for the familiar task condition where Hypothesis 2 but not Hypothesis 1 predicts development across task blocks. In the pseudoword learning task, the predictions of the two hypotheses do not differ.

In addition to testing these hypotheses regarding the effects of novelty and task demands on strategy use, we were also interested in replicating our previous strategy-related findings. In a recent study, we examined the evolvement of strategy use and objective task performance in an adaptive n-back task that was novel to the participants (Waris et al., 2021). The results of that study showed that (1) about half of the participants (51.5% in Experiment 1 and 52.7% in Experiment 2) reported strategy use already during the very first task block, (2) strategy use increased and became more stable during the initial task blocks, (3) strategy type as well as the level of detail in the open-ended strategy reports were associated with objective task performance, and (4) open-ended versus multiple-choice strategy questions gave partly discrepant results. To probe the robustness and generality of these previous findings, we wanted to replicate them with another novel task, namely the pseudoword list learning task.

The relevance of strategies for understanding memory performance has become clear in previous research where a considerable portion of participants have reported using different learning strategies spontaneously when performing episodic memory tasks, and where different memory strategies have been associated with task performance, depending on the type of task (e.g., Camp et al., 1983; Hill et al., 1990; McDaniel & Kearney, 1984). A distinct feature of these previous studies, and this one as well, is the probing of participants’ memory strategy use through self-reports. Strategy use during task performance can be measured with open-ended queries, list-based queries containing printed descriptions of different strategies, or by inferring from objective performance variables (e.g., by observing patterns in response tendencies or reaction times during recall). We recently found that repeated list-based strategy queries, which contain printed descriptions of different strategies, affect participants’ task performance (Waris et al., 2021). However, it is unclear whether repeated open-ended strategy queries also affect task performance. Hypothetically, this could occur if the presentation of open-ended queries affects participants’ metacognitive processing or demand characteristics, resulting in development of more efficient strategies and better performance. This methodological issue was tested by including a control group that only responded to the strategy queries after completing both word-list learning tasks.

Thus, our study had three main aims: (1) to test two hypotheses regarding the effects of novelty and task difficulty on strategy use, (2) to replicate our previous results regarding strategy use in a novel working memory task, and (3) to probe whether repeated open-ended strategy queries affect task performance and/or strategy use. When assessing each aim, we refrained from using null hypothesis significance testing, and instead employed the recently recommended Bayesian Inference (Jeffreys, 1961; Kass & Raftery, 1995), having the possibility to contest our hypotheses in both directions with a strength of evidence on an continuous scale (Schönbrodt & Wagenmakers, 2018; Wagenmakers et al., 2018). Moreover, due to our block-level data with five different time points, we employed linear mixed effects (LME) modelling (Baayen et al., 2008) whenever applicable. LME can take into account variance from different sources (random and fixed effects), and it is considered more appropriate than traditional analyses of variance (ANOVAs) when investigating change over time (Mirman, 2014), which is critical in this study.

## Methods

### Ethics statement

This pre-registered study (https://osf.io/24tcy) was approved by the Joint Ethics Committee at the Departments of Psychology and Logopedics, Åbo Akademi University. All participants provided their informed consent separately for both the prescreening study and the study proper (see below), participation was anonymous, and the participants were informed of their right to withdraw their participation at any time during the study.

### Participants and procedure

The study consisted of a separate prescreening study and the study proper. Participants were recruited via the Prolific (https://www.prolific.co/) crowdsourcing site. The study was administered online using our in-house web-based test platform that employs a domain-specific programming language tailored to building psychological tasks. Participants who took part in the prescreening study were not informed of the fact that it served as a screen for the actual study. The screening study was estimated to take approximately 12 min in total, and the participants were paid £1.20 (approximately US$1.60 at the time) upon completion. The study proper was estimated to take approximately 30 min, and the participants were paid £3 (approximately US$3.9 at the time) upon completion.

#### The prescreening study

The prescreening study included a background questionnaire, a simple picture description task tapping verbal productivity (Waris et al., 2021), and select personality measures.^1^ For the prescreening study, we implemented Prolific’s built-in screening tool to invite 18- to 50-year-old participants whose first language was English and nationality had been marked as the United Kingdom, the United States, Ireland, Australia, Canada, or New Zealand. Furthermore, according to self-reports, participants had not been diagnosed with dyslexia, dyspraxia, attention-deficit hyperactivity disorder (ADHD), or literacy difficulties, and they had no diagnosed uncontrolled mental health condition that significantly impacted their daily life. Altogether 505 participants were prescreened in three separate waves (pilot wave, *n* = 23; Wave 1, *n* = 384; Wave 2, *n* = 98). Note that the second wave was conducted to fulfil the pre-registered group quotas for the study proper, and therefore, only a limited number of participants from that wave could participate in the actual study.

#### The study proper

Following our pre-registered inclusion criteria, prescreened participants were invited to the actual study if: their first language was English, they had no neurologic or psychiatric illness that affected their life, they had not been diagnosed with a neurodevelopmental disorder (e.g., dyslexia, ADHD), they had no self-reported trouble reading the questionnaire items, they used no central nervous system (CNS)-active medication(s) or drugs (except tobacco, alcohol, and cannabis), they had not consumed more than nine units of alcohol on the previous day, they were not intoxicated at the time of testing, they passed all three attention checks, and their picture description was not deemed as irrelevant or lacking any actual detail (e.g., “that’s nice”).^2^ In addition, two participants could not be invited as they had provided ambiguous participant IDs. Out of the 505 prescreened participants, 357 (70.7%) were invited to the actual study.

The study proper included a selection of background questions, the word and pseudoword list learning tasks, the Working Memory Questionnaire (Vallat-Azouvi et al., 2012), a modified version of the Internal Memory Aids questionnaire (Chouliara & Lincoln, 2015), a posttest strategy questionnaire, and some posttest questions. Participants were randomised into one of four groups. Two groups gave open-ended strategy reports each time they had recalled items for the to-be-learned item list (i.e., after each task block, altogether five times), while the other two groups only received separate open-ended strategy queries for each task after they had completed both tasks. For counterbalancing purposes, in both group pairs one group received the word list task with real words first, and the pseudoword task second, while the task order was reversed for the other group. As the task order did not affect performance (see section Effect of task order and word list variants on recall performance), it was not included in the analyses.

The study proper was completed by 195 participants. For the pretest background items, the same inclusion criteria applied as for the prescreening study, and additionally, the participants had to recall at least a total of 10 real words and 5 pseudowords in the 2 tasks, report receiving/using no external aids (e.g., note-taking) to solve either word list task, report no previous experience with a comparable pseudoword list learning task, and have no discrepant responses in the prescreening and the actual study on the background items regarding age (+1 year allowed), gender, and education (highest attained degree, ±1 level in education allowed^3^). Twenty-five participants were excluded on the basis of these pre-defined exclusion criteria. Moreover, we observed nine corrupted strategy responses^4^ when coding the open-ended queries, which resulted in a final sample size of 161 participants (Group receiving repeated strategy queries, *n* = 101; Group receiving single strategy queries, *n* = 60). See Table 1 for the participants’ background characteristics.

**Table 1.** Background characteristics in the two groups.

	RSQ Group	SSQ Group
Sample size (*n*)	101	60
Gender (F/M)	72/29	36/24
Age (*M, SD*)	34.75 (8.45)	32.48 (9.14)
Education	Lower secondary 2.0%	Lower secondary 5.0%
	Higher secondary 19.8%	Higher secondary 20.0%
	Basic vocational 3.0%	Basic vocational 5.0%
	Vocational university 9.9%	Vocational university 18.3%
	Bachelor’s degree 47.5%	Bachelor’s degree 38.3%
	Master’s degree 14.9%	Master’s degree 10.0%
	Doctoral degree 3.0%	Doctoral degree 1.7%

RSQ: Repeated Strategy Queries; SSQ: Single Strategy Queries.

### List learning task with real words

The list learning task with real words consisted of an 18-word list (see Table 2) that was presented five times. The order of the to-be-learned items was randomised every time the list was presented. The participants were instructed to memorise as many words as possible irrespective of order. Two separate lists were used to minimise the risk that the observed results would be list-specific.

**Table 2.** Both versions of each stimulus list in the word and pseudoword list learning tasks.

Real words List 1	PALACE, ISLAND, STREET, HILL, POCKET, SISTER, TRASH, SOAP, TOOTH, POOL, TOWER, FLOWER, RING, NEEDLE, SWEAT, BOOT, HAWK, BLOOD
Real words List 2	BABY, NOSE, TENNIS, PERSON, BOWL, WALLET, SWORD, RECORD, HEART, PAINT, PIANO, SINK, APPLE, POLE, BRIDGE, GLOVE, RAIN, LAKE
Pseudowords List 1	ENKS, TRODE, DOUD, FLINCE, PRANTS, ZOARS, MEPHED, RANS, GREPT, ECSED
Pseudowords List 2	NIRS, CHAIZE, TOARD, SOLDE, STRUYS, BROORS, GROIZ, PENX, NOST, PALD

We used the MRC Psycholinguistic Database (Coltheart, 1981) to select words that: (1) were nouns according to the SOED database (Dolby et al., 1963), (2) were 4–6 letters long, (3) consisted of 1–3 syllables, (4) had a Kučera-Francis (Kučera & Francis, 1967) written frequency above 0, and (5) had concreteness and imageability ratings of 558 or more (i.e., at least 1 *SD* above the mean). This gave us a pool of 444 words. Next, using the SUBTLEX-US corpus (Brysbaert & New, 2009), we narrowed down the pool to the 283 high-frequency words as defined by Zipf frequency values of four or more (van Heuven et al., 2014). From this final pool, two lists of 18 common nouns were randomly selected. We ran independent samples *t*-tests to test whether the lists differed significantly in the above-mentioned variables and settled on the lists when all *t*-tests resulted in *p*-values of .3 or higher, number of letters, *t*(34) = 0.59, *p* = .56, *d* = 0.20; number of phonemes, *t*(34) = 0.20, *p* = .84, *d* = 0.06; number of syllables, *t*(34) = 0.00, *p* = 1.00, *d* = 0.00; concreteness, *t*(34) = 0.03, *p* = .98, *d* = 0.01; imageability, *t*(34) = 0.54, *p* = .59, *d* = 0.18; Kučera-Francis written frequency, *t*(34) = 0.04, *p* = .97, *d* = 0.01; Zipf frequency value, *t*(34) = 0.65, *p* = .52, *d* = 0.22.

The words were shown on-screen for 1 s, and they were separated by a 1-s blank-screen interval. After the final word in a list had been shown, a distractor task appeared on-screen (presented as an “Attention check”). The distractors were arithmetical tasks (e.g., 9 + 8 – 7 + 6 = ?) that were intended to be relatively simple but still demanding enough to effectively replace the to-be-remembered words from working memory and thus diminish the contribution of short-term storage to task performance. One was to select the correct alternative for the distractor tasks among five options. The participants were required to get 3/5 distractor items correct to be included in the statistical analyses.

After the distractor task, the response screen was displayed. It included short instructions and a set of 18 boxes in three columns. The participants were instructed to type in the words they could recall one at a time in any order in the response boxes. After typing a word, the participants were instructed to click on a green “Next word” box to type in another word. When the participants had finished recalling words for a given list, they were to click on a red “I am done recalling words for this list” box. Words had to be typed correctly, but non-letter characters or spaces were permitted before or after the word. The primary dependent measure was the number of correctly recalled words per list.

After recalling words for each list, two of the groups received open-ended strategy queries, while two of the groups received these queries not until they had completed both list learning tasks. The exact phrasing of the open-ended query was “Please describe in as much detail as possible how you solved the previous word list task (not the math task). That is, how did you try to memorize the words?”

### List learning task with pseudowords

The pseudoword list learning task was identical to the real word task, except that the stimuli were pseudowords (e.g., doud, rans) and that the list contained 10 items instead of 18 (see Table 2).

We used the ARC Nonword Database (Rastle et al., 2002) to select an initial item pool that was similar to the pool of real words in that it contained 120 items with four letters, 94 words with five letters, and 69 words with six letters. When searching for items, we used the following search criteria: (1) only legal bigrams, (2) morphologically ambiguous syllables, (3) neighbourhood size: one or greater, (4) summed frequency of neighbours: one or greater, (5) bigram frequency (position nonspecific)—token: one or greater, (6) trigram frequency (position nonspecific)—token: one or greater, and (7) number of phonemes: 1–6. From the pool of items, we randomly selected 20 pseudowords that were randomised into two lists. We ran independent samples *t*-tests to test whether the lists differed significantly in the above-mentioned variables, and we settled on the lists when all *t*-tests resulted in *p*-values of .3 or higher, number of letters, *t*(18) = 0.26, *p* = .80, *d* = 0.12; neighbourhood size, *t*(18) = 0.23, *p* = .82, *d* = 0.10; summed frequency of neighbours, *t*(18) = 0.50, *p* = .63, *d* = 0.22; bigram frequency, *t*(18) = 0.92, *p* = .37, *d* = 0.41; trigram frequency, *t*(18) = 0.04, *p* = .97, *d* = 0.02; number of phonemes, *t*(18) = 0.81, *p* = .43, *d* = 0.36.

### Rating participants’ strategy descriptions

Two independent raters classified each strategy report into 1 of 12 different types, which were identical to those asked in the list-based query at the end of the study: (1) Rehearsal/Repetition (actively repeating the items aloud or in mind), (2) Grouping into larger units, (3) Visualisation (mentally seeing what the items might represent), (4) Spatial association (e.g., placing what the items represent on a street), (5) Semantic/verbal association (with real words; grouping words together based on their meaning; with pseudowords; associating the pseudowords with real words), (6) Narrative (creating a story), (7) Instinct, (8) Selective focus (focusing only on a subset of the items), (9) Guessing, (10) Other strategy, (11) No strategy, and (12) Did not understand. If the participants named more than one strategy, they were coded as secondary, tertiary, and so on, based on the order in which they were reported. The primary strategies (i.e., those named as first) were used in the analyses.

Besides the more detailed strategy categories, we examined strategy use also by employing a broader 4-category classification of strategies taken from Fellman et al. (2020; see also Camp et al., 1983). Here, the strategies Rehearsal/Repetition and Selective focus were classified as a Maintenance strategy, whereas Grouping, Visualisation, Spatial association, Verbal/Semantic association, and Narrative were classified as a Manipulation strategy. Instinct, Guessing, No strategy, and Did not understand were classified as No strategy, and all the other strategies were tallied under Other strategy.

In addition to strategy types, we coded the level of detail (LoD) in the open-ended responses (cf. Fellman et al., 2020; Forsberg et al., 2020; Laine et al., 2018; Waris et al., 2021). A *detail* was defined as either a report of a specific strategy (STR) or of a specific strategy feature (FT). In practice, features did not occur without an associated specific strategy. Thus, 0 points were given for no reported detail; 1 point was given for one detail (in practice, this was always a STR; for example, “I remembered the words in groups of two [Grouping]”); 2 points was given for (2 STR; for example, “I created a story [Narrative] by focusing on only some of the words [Selective focus]”) or (1 STR and 1 FT; for example, “I grouped the items into categories [Semantic association], like furniture and animals”); 3 points were given to (3 STR) or (1 STR + 2 FT) or (2 STR + 1 FT); and 4 points (maximum) were given to (4 STR) or (1 STR + 3 FT) or (2 STR + 2 FT) or (3 STR + 1 FT) or above.

After the independent coding was performed, we ran reliability analyses of the ratings. For the classification of strategy reports into the strategy types, the unweighted kappa coefficients concerning the pseudoword condition and the real word condition were κ = 0.86, and κ = 0.78, respectively. For the scoring of the level of detail in the strategy reports, the weighted kappa coefficients in the pseudoword and the real words conditions were κ_w_ = .0.74, and κ_w_ = 0.72, respectively. All the reliability coefficients were considered acceptable, and hence, the raters proceeded to consensus decisions for diverging strategy classifications and level of detail scores.

### The posttest strategy questionnaire

The posttest strategy questionnaire was presented after each participant had completed both list learning tasks and answered all open-ended strategy queries (see Supplementary material for strategy questionnaires). It consisted of three questions for each list learning task. First, the participants were to indicate which strategy or strategies they had used in the last (fifth) block of the respective list learning task. Next, they were to indicate what strategy they had used most often in the last block, and finally, they were to select what strategy, of the ones they had used, they felt was the most sophisticated one. The strategy questions were presented in the same order as the participant had completed the tasks (pseudoword-then-word learning or vice versa).

### Analytical approach

In this study, we employed Bayesian factors (BFs) to test our pre-defined hypotheses, using the “BayesFactor” package (Morey & Rouder, 2015) in R version 3.5.2. (R Core Team, 2018). With this analytical approach, the evidence for either the null hypothesis (H_01_) or for the alternative hypothesis (H_10_) is contested on a continuous scale. A BF of 1 indicates perfect ambiguity (i.e., no evidence for either hypothesis), whereas a BF above or below 1 provides evidence for the H_01_ or H_10_, respectively. For the interpretation of the BFs, we followed the guidelines proposed by Kass and Raftery (1995), where BFs between 1 and 3 are defined as “weak evidence,” BFs between 3 and 20 as “positive evidence,” BFs between 20 and 150 as “strong evidence,” and BFs >150 as “very strong evidence.” Besides BFs, we also report estimates of between-group mean differences using a posterior distribution with 10,000 iterations coupled with their 95% credible intervals (see, for example, De Simoni & von Bastian, 2018) formed from the highest density interval (HDI) distribution. In each BF analysis, we used the default prior setting (i.e., Cauchy distribution using a scaling factor *r* = .707). Given that our episodic memory tasks consisted of five consecutive blocks, we chose to employ LME (Baayen et al., 2008) models whenever possible. In these models, participants were treated as the crossed-random effect, while Block (coded as a linear contrast) always served as one of the fixed effects. Thus, these LME models let us investigate change over time (Mirman, 2014), which is critical in this study.

As regards outlier analysis, we screened task performances for univariate outliers using the summed proportion score across blocks as the dependent variable separately for the performance in the pseudoword task and the real word task. Univariate outliers were defined as those scoring three times the interquartile range above or below the first or the third quartile in each task. In this study, no such outliers were identified.

### Effect of task order and word list variants on recall performance

For assuring that the order in which the participants (*n* = 161) received the two task conditions (pseudoword and real word) did not affect task performance, we employed an LME model. It showed positive evidence against a main effect of task order (*M*_diff_ = 0.12, 95% HDI = [−0.18, 0.40], BF_01_ = 16.67 ± 6.64%), indicating that the task order did not affect task performance. We also obtained strong evidence against an Order × Block interaction (*M*_diff_ = −0.09, 95% HDI = [−0.37, 0.18], BF_01_ = 16.67 ± 2.99%), indicating that the task order had no impact on the performance improvements across blocks. The same LME model was employed for testing the list variants. It showed positive evidence against a main effect of list (*M*_diff_ = 0.35, 95% HDI = [0.05, 0.63], BF_01_ = 9.09 ± 1.32%), and strong evidence against a List × Block interaction (*M*_diff_ = −0.08, 95% HDI = [−0.36, 0.19], BF_01_ = 16.67 ± 1.81%).

## Results

Data and R code for all analyses can be found on the Open Science Framework at https://osf.io/udse4/. Given the number of specific predictions and research questions examined, we refer the reader to Table 6 at the end of the Results for an overview of the pattern of results. The present chapter is structured as follows. Section “Learning progress” presents the learning curves in the two task conditions. Sections “Strategy development: frequency of strategy use across the task blocks,” “Strategy development: level of strategy detail across the task blocks,” “Relationships between strategy type and episodic memory performance,” and “Relationships between strategy LoD and episodic memory performance” test the predictions derived from the two hypotheses. These had been pre-registered and were presented in the Introduction. Section “Replication of our earlier findings on strategy development in a working memory updating task” reports the replication attempt of our previous results regarding strategy use in a novel memory task. The earlier findings we attempted to replicate overlap partly with the predictions of our two hypotheses. Finally, section “The influence of repeated open-ended strategy queries on task performance and strategy use” examines whether repeated open-ended strategy queries affect task performance and/or strategy use.

### Learning progress

**Figure 1.**
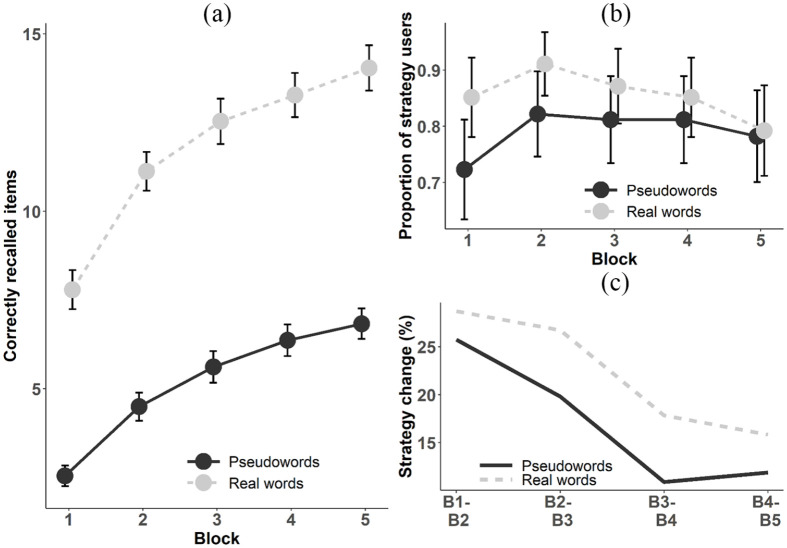
(a) Proportion of correctly recalled items by condition across blocks. (b) Proportion of strategy users by condition across blocks according to participants’ open-ended strategy reports. (c) Strategy change from one block (B) to another by condition across blocks. Note that those receiving strategy queries by the end of the session have been excluded (thus *n* = 101). Error bars represent 95% confidence intervals.

The number of correctly recalled items per block in the word and pseudoword learning conditions is presented in Figure 1(a). As expected, the results showed very strong evidence for a main effect of block both in the pseudoword condition (*M*_diff_ = 2.09, 95% HDI = [1.94, 2.23], BF_10_ > 150 ± 0.65%) and the real word condition (*M*_diff_ = 2.92, 95% HDI = [2.65, 3.16], BF_10_ > 150 ± 0.46%), indicating that the performance improved across the task blocks. However, as depicted in Figure 1(a), the learning curve remained clearly lower in the novel pseudoword condition.

### Strategy development: frequency of strategy use across the task blocks

Hypothesis 1 (novelty hypothesis) predicts that only the pseudoword condition shows increase in the frequency of strategy use, while Hypothesis 2 (task demand hypothesis) assumes that strategy use increases over time in both task conditions. Strategy use was prevalent in both tasks, as the majority of the participants reported using a strategy already in the first block (see Figure 1(b) and Table 3). A two-way Block × Condition LME model where strategy frequency was coded as a proportional dependent variable (0 = *did not use a strategy*; 1 = *used a strategy*) revealed strong evidence for a main effect of condition, as the frequency of strategy use in the real word condition was overall higher than in the pseudoword condition (*M*_diff_ = −0.03, 95% HDI = [−0.05, 0.00], BF_10_ = 63.16 ± 2.0%) (see also Figure 1(b)). We observed positive evidence against a main effect of block (*M*_diff_ = −0.01, 95% HDI = [−0.03, 0.02], BF_01_ = 12.50 ± 1.72%), indicating that overall, strategy use did not change across the five blocks, which goes against both hypotheses. This was modified by weak evidence for a Block × Condition interaction, with a somewhat higher frequency of strategy use in the real word condition in the early but not the later parts of the task (*M*_diff_ = 0.06, 95% HDI = [0.01, 0.10], BF_10_ = 1.74 ± 2.03%), being once again against the hypotheses.

**Table 3.** Proportion (%) of participants using different strategy types across blocks in the two list learning conditions.

Strategy		Block 1	Block 2	Block 3	Block 4	Block 5
		%	%	%	%	%
General	Specific	Pseudoword learning task				
No	No strategy	27.72	17.82	18.81	18.81	20.79
	Guessing	0	0	0	0	0.99
Maintenance	Rehearsal/Repetition	32.67	28.71	31.68	30.69	31.68
	Selective focus	7.92	21.78	23.76	25.74	18.81
Other	Other strategy	8.91	9.9	6.93	6.93	7.92
Manipulation	Grouping	0	0	0.99	0.99	0.99
	Narrative	0.99	0.99	0.99	0.99	1.98
	Verbal/Semantic association	19.8	18.81	15.84	13.86	14.85
	Visualisation	1.98	1.98	0.99	1.98	1.98
		Real word learning task				
No	No strategy	14.85	8.91	12.87	14.85	20.79
Maintenance	Rehearsal/Repetition	32.67	32.67	31.68	26.73	28.71
	Selective focus	6.93	21.78	20.79	18.81	12.87
Other	Other strategy	12.87	9.9	6.93	7.92	7.92
Manipulation	Grouping	1.98	2.97	5.94	8.91	8.91
	Narrative	8.91	7.92	7.92	8.91	8.91
	Verbal/Semantic association	5.94	4.95	4.95	3.96	5.94
	Visualisation	15.84	10.89	8.91	9.9	5.94

*Note*. Only those participants receiving strategy queries following each block are included. Strategies that are not listed were not reported by any participants. *N* = 101.

As our earlier block-by-block strategy findings with a memory task (Waris et al., 2021) showed a very early increase in strategy use from block 1 to block 2 followed by a rather flat curve, we also analysed strategy increases from the first block to the second block. These results showed strong evidence for a main effect of condition (*M*_diff_ = −0.13, 95% HDI = [−0.19, −0.07], BF_10_ = 83.21 ± 3.49%), as strategy use in the first two blocks appeared more often in the real word condition compared with the pseudoword condition. Now we obtained positive evidence for a main effect of block (*M*_diff_ = 0.08, 95% HDI = [0.02, 0.13], BF_10_ = 3.98 ± 3.98%), which indicated increased strategy use from the first block to the second one. The results showed positive evidence against a Condition × Block interaction (*M*_diff_ = 0.04, 95% HDI = [−0.07, 0.15], BF_01_ = 4.55 ± 3.09%).

In sum, while frequency of strategy use did not indicate a change when the whole task sequences were analysed, a subsequent analysis on the first two blocks showed an increase in strategy use between blocks 1 and 2 that was not modulated by task condition. This initial strategy development is in line with Hypothesis 2 which predicts increased strategy use in both task conditions.

### Strategy development: level of strategy detail across the task blocks

**Figure 2.**
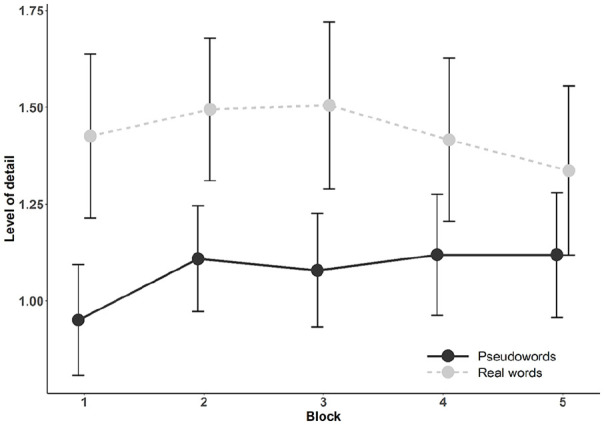
Average level of strategy detail per task block. Note that those receiving strategy queries by the end of the session are excluded (thus *n* = 101). Error bars represent 95% confidence intervals.

Hypothesis 1 (novelty hypothesis) predicts an increase in LoD only in the pseudoword condition, while Hypothesis 2 (task demand hypothesis) assumes that LoD increases over time in both task conditions. First, analysing LoD over all blocks (see Figure 2), we observed very strong evidence for a main effect of condition (*M*_diff_ = −0.36, 95% HDI = [−0.40, −0.32], BF_10_ > 150 ± 2.13%), indicating that the LoD scores were higher throughout the task in the real word condition, compared with the pseudoword condition. We found positive evidence against a main effect of block (*M*_diff_ = 0.01, 95% HDI = [−0.05, 0.07], BF_01_ = 14.29 ± 2.09%), indicating that LoD did not increase when the whole task sequence was taken into account. We obtained weak evidence against a Block × Condition interaction (*M*_diff_ = 0.12, 95% HDI = [0.00, 0.23], BF_01_ = 1.11 ± 2.09%).

Following the LoD results of Waris et al. (2021) that showed the highest increase in LoD between the first two task blocks after which the curve evened out, we analysed the changes from block 1 to block 2 also for LoD. The results showed very strong evidence for a main effect of condition (*M*_diff_ = −0.43, 95% HDI = [−0.55, −0.29], BF_10_ > 150 ± 8.26%), indicating that LoD levels were higher in the real word condition compared with the pseudoword condition during the two initial blocks. We obtained weak evidence against a main effect of block (*M*_diff_ = 0.11, 95% HDI = [−0.02, 0.24], BF_01_ = 1.96 ± 8.18%), suggesting that LoD did not increase from the first block to the second block. The results showed positive evidence against a Condition × Block interaction (*M*_diff_ = 0.08, 95% HDI = [−0.16, 0.33], BF_01_ = 5.26 ± 8.26%).

To sum up, the LoD results over time did not support either of the two hypotheses, as they did not increase across task blocks in either condition. This was true both for the analysis covering all blocks and for the analysis focusing on the first two blocks.

### Relationships between strategy type and episodic memory performance

Both Hypothesis 1 (novelty hypothesis) and Hypothesis 2 (task demand hypothesis) assume that, irrespective of task condition, objective recall performance across blocks is positively associated with strategy use, including strategy type. In the present analyses, we examined associations between strategy use and memory performance separately in the pseudoword and the real word learning conditions by grouping the participants based on their open-ended strategy reports. For this, we used the broader categories described above (Maintenance, Manipulation, Other strategy, No strategy), but followed Fellman et al. (2020) by lumping together Maintenance and Other, which resulted in three distinct strategy categories. The participants were grouped according to what strategy category (Maintenance/Other, Manipulation, No strategy) they had reported using most frequently during each task. If a participant reported using two different strategies an equal number of times, the more sophisticated strategy was chosen (No strategy < Maintenance/Other < Manipulation; see also Fellman et al., 2020). This resulted in relatively balanced groups in both task conditions (pseudoword condition: No strategy, *n* = 21; Maintenance/Other, *n* = 61; Manipulation, *n* = 19; real word condition: No strategy, *n* = 12; Maintenance/Other, *n* = 59; Manipulation, *n* = 30).

LME models were computed to test whether strategy type was associated with episodic memory performance across blocks. We performed pairwise comparisons between each strategy type (see Table 4 and Figure 2). In *the pseudoword condition*, we observed weak evidence for a main effect of strategy between Manipulation and No strategy (*M*_diff_ = 1.06, 95% HDI = [−0.01, 2.23], BF_10_ = 2.40 ± 2.04%) and between Maintenance/Other strategy and No strategy (*M*_diff_ = 0.92, 95% HDI = [0.05, 1.79], BF_10_ = 2.58 ± 2.98%), both suggesting worse recall performance for No strategy. Besides that, BF supported the null hypothesis in the other main effects and Strategy × Block interactions in the pseudoword condition (see Table 4).

**Table 4.** Pairwise comparisons between the most used strategies on episodic memory performance based on the repeated strategy queries.

Effect	MNP vs MNT/OTHR^a^		MNP vs NS^a^		MNT/OTHR vs NS^b^	
	*M*_Diff_ [95% HDI]	BF ± error (%)	*M*_Diff_ [95% HDI]	BF ± error (%)	*M*_Diff_ [95% HDI]	BF ± error (%)
Pseudowords						
Strategy	0.2 [−0.2, 0.6]	BF_01_ = 3.12 ± 2.4	1.06 [0.01, 2.23]	BF_10_ = 2.40 ± 2.04	0.92 [0.05, 1.79]	BF_10_ = 2.58 ± 2.98
Block	2.12 [1.93, 2.32]	**BF**_10_ **>** **150** **±** **2.27**	2.01 [1.78, 2.23]	**BF**_10_ **>** **150** ± **1.98**	2.02 [1.84, 2.21]	****BF**_10_ ** **>** **150** **±** **3.01**
Interaction	0.01 [−0.19, 0.2]	BF_01_ = 20 ± 2.2	0.21 [−0.01, 0.43]	BF_01_ = 9.09 ± 2.27	0.2 [0.01, 0.38]	BF_01_ = 11.11 4.3
Real words						
Strategy	1.25 [0.76, 1.78]	**BF**_10_ = **3.73** **±** **1.34**	3.58 [2.75, 4.44]	**BF**_10_ **>** **150** **±** **1.25**	2.26 [1.56, 2.94]	****BF**_10_**= **44.4** **±** **1.4**
Block	3.1 [2.85, 3.37]	**BF**_10_ **>** **150** **±** **1.34**	2.46 [2.01, 2.87]	**BF**_10_ **>** **150** **±** **1.3**	2.37 [1.96, 2.76]	****BF**_10_ ** **>** **150** **±** **1.56**
Interaction	0.2 [−0.05, 0.47]	BF_01_ = 14.29 **±** 2.46	1.45 [1.03, 1.88]	**BF**_10_ = **20.96** **±** **14.03**	1.26 [0.86, 1.64]	**BF**_10_ = **8.52** **±** **2.0**

MNP: Manipulation; MNT/OTHR: Maintenance or Other strategy; NS: No strategy; HDI: highest density interval of the posterior distribution; BF: Bayesian factor.

Estimates are the mean group differences from 10,000 samples of the posterior distribution.

a Positive values represent greater performance in the MNP.

b Positive values represent greater performance in the MNT/OTHR.Bolded values are the results that provide evidence for the alternative hypothesis.

In *the real word condition*, we observed positive evidence for a main effect of strategy between Manipulation and Maintenance/Other strategy (*M*_diff_ = 1.25, 95% HDI = [0.76, 1.78], BF_10_ = 3.73 ± 1.34%), indicating that those using manipulation strategies in the real word condition performed better across the task. We obtained positive evidence against an interaction effect between Manipulation and Maintenance/Other strategy and block (*M*_diff_ = 0.20, 95% HDI = [−0.05, 0.47], BF_01_ = 14.29 ± 2.46%). As regards the pairwise comparison between Manipulation and No strategy use, we observed very strong evidence for a main effect of strategy (*M*_diff_ = 3.58, 95% HDI = [2.75, 4.44], BF_10_ > 150 ± 1.25%) as well as positive evidence of an interaction effect (*M*_diff_ = 1.45, 95% HDI = [1.03, 1.88], BF_10_ = 20.96 ± 14.03%). This indicated that Manipulation, as compared with no strategy use, resulted in a better overall episodic memory performance and steeper learning curves across blocks in the real word condition. The same pattern was observed between those using Maintenance/Other strategies compared with No strategy, where we observed strong evidence for a main effect of strategy (*M*_diff_ = 2.26, 95% HDI = [1.56, 2.94], BF_10_ = 44.4 ± 1.95%) and positive evidence for a Strategy × Block interaction (*M*_diff_ = 1.26, 95% HDI = [0.86, 1.64], BF_10_ = 8.52 ± 2.0%).

**Figure 3.**
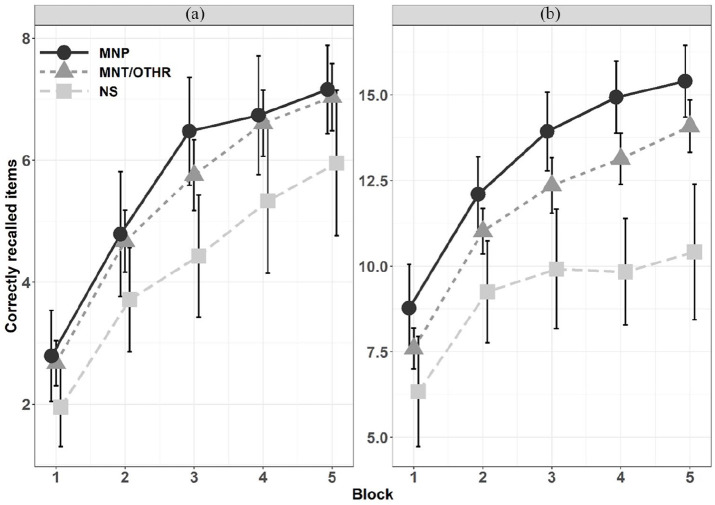
Episodic memory performance in the (a) pseudoword condition and the (b) real word condition as a function of the most commonly used strategy type across blocks. MNP: Manipulation; MNT/OTHR: Maintenance or Other strategy; NS: No strategy. Error bars represent 95% confidence intervals.

In sum, the analyses on the real word condition described above provide evidence for a positive relationship between strategy use and episodic memory performance as predicted by both hypotheses. However, this evidence was lacking for the pseudoword condition, albeit the overall pattern in Figure 3 looked partly similar as with the real words.

### Relationships between strategy LoD and episodic memory performance

It is assumed by both hypotheses that, irrespective of task condition, objective recall performance across blocks is positively associated with strategy LoD (see Figure 2). We examined the association between LoD and episodic memory performance separately in the pseudoword and real word condition using Bayesian LME models. The results showed very strong evidence for a main effect of LoD both in the pseudoword condition (*M*_diff_ = 0.51, 95% HDI = [0.28, 0.74], BF_10_ > 150 ± 1.08%) and the real word condition (*M*_diff_ = 0.78, 95% HDI = [0.51, 1.05], BF_10_ > 150 ± 1.19%), indicating that those those with higher LoD scores had overall better episodic memory performance. There was positive evidence against a Block × LoD interaction in both the pseudoword condition (*M*_diff_ = −0.01, 95% HDI = [–0.21, 0.20], BF_01_ = 16.67 ± 1.47%) and in the real word condition (*M*_diff_ = 0.05, 95% HDI = [−0.20, 0.28], BF_01_ = 14.29 ± 1.82%), indicating that the LoD effect on recall performance did not change over time. These results are in line with both hypotheses by revealing an association between LoD and recall performance in both task conditions.

### Replication of our earlier findings on strategy development in a working memory updating task

Our previous results on within-task strategy development (Waris et al., 2021) stem from a different memory task (n-back working memory updating task), and we wanted to examine whether another type of novel memory task (pseudoword learning) elicits a similar pattern of strategy deployment. After all, the cognitive skill learning approach presupposes that reactions to task novelty follow common stages (e.g., Chein & Schneider, 2012).

The findings from the study by Waris et al. (2021) that we wanted to replicate were as follows: (1) about half of the participants reported strategy use already during the very first task block, (2) strategy use increased and became more stable during the initial task blocks, (3) strategy type as well as the level of detail of the open-ended strategy reports were associated with objective task performance, and (4) open-ended and multiple-choice strategy questions gave inconsistent results.

As can be seen in Figure 1(b), point (1) was replicated, as even in the present study over half of the participants reported strategy use already in the very first task block of the pseudoword learning task. In other words, from a very early stage, spontaneous strategy use appears to be a predominant aspect of novel memory task performance.

As regards point (2), the analysis in section “Strategy development: frequency of strategy use across the task blocks” that focused on the first two task blocks showed that the frequency of strategy use increased from block 1 to block 2, thus replicating the finding by Waris et al. (2021) for the initial part of the task (see Figure 1(b)). Regarding stability of strategy use, Figure 1(c) depicts the rates of participants who changed their strategy from one block to another. Again, here as well as in the study of Waris et al., change in strategy was most common within the first blocks of the task, thus replicating the earlier finding.

Point (3) was addressed in sections “Relationships between strategy type and episodic memory performance” and “Relationships between strategy LoD and episodic memory performance.” The analyses presented in these sections partly replicated the association between strategy use and objective performance, as strategy level of detail was associated with objective performance, but strategy type was not.

As regards point (4), this study as well as that of Waris et al. (2021) indicated quite similar discrepancy rates between the list-based and open-ended queries (55.3% agreement in the present pseudoword task and 52.3% and 50% agreement rates in the earlier study employing a working memory task). In other words, also this finding by Waris et al. (2021) was replicated.

In summary, all four findings from Waris et al. (2021) found support in this study.

### The influence of repeated open-ended strategy queries on task performance and strategy use

To explore this question, we first investigated whether the group giving strategy reports throughout the blocks (Repeated Strategy Queries, RSQ) differed in objective recall performance when compared with the control group that only gave a single strategy report that was completed after both list learning tasks had been finished (Single Strategy Queries, SSQ). We explored this with an LME three-way interaction analysis with Block, Condition, and Group as predictors (see Table 5 that summarises the outcomes). The results showed very strong evidence of a Block × Condition interaction (*M*_diff_ = −0.85, 95% HDI = [−0.98, −0.71], BF_10_ > 150 ± 8.33%), indicating that the performance increased more across blocks in the real word condition, compared with the pseudoword condition. Importantly, the results showed positive evidence against a Block × Group interaction (*M*_diff_ = −0.09, 95% HDI = [−0.23, 0.04], BF_01_ = 20.00 ± 9.40%), indicating that the two groups’ episodic memory performance improved to the same extent across task blocks. In line with this, we observed positive evidence against a Block × Group × Condition interaction (*M*_diff_ = 0.01, 95% HDI = [−0.12, 0.15], BF_01_ = 20.0 ± 9.34%). Thus, irrespective of condition (i.e., pseudoword learning vs real word learning), the RSQ and SSQ groups showed similar performance improvements across blocks.

**Table 5.** Results from the LME model with all main effects, two-way, and three-way interactions.

Effect	*M_diff_*	Lower HDI	Upper HDI	BF ± error %
Block	2.46	2.32	2.59	**BF**_10_ **>** **150** **±** **0.66**
Condition	−6.68	−6.78	−6.58	**BF**_10_ **>** **150** **±** **1.45**
Group	−0.19	−0.49	0.09	BF_01_ = 14.29 ± 1.21
Block × Condition	−0.85	−0.98	−0.71	**BF**_10_ **>** **150** **±** **6.64**
Block × Group	−0.09	−0.23	0.04	BF_01_ = 20 ± 9.4
Condition × Group	−0.09	−0.19	0.00	BF_01_ = 11.11 ± 6.09
Block × Condition × Group	0.01	−0.12	0.15	BF_01_ = 25 ± 6.1

LME: linear mixed effects; HDI: highest density interval of the posterior distribution; BF: Bayesian factor.

Estimates are the mean group differences from 10,000 samples of the posterior distribution.Bolded values are the results that provide evidence for the alternative hypothesis.

**Figure 4.**
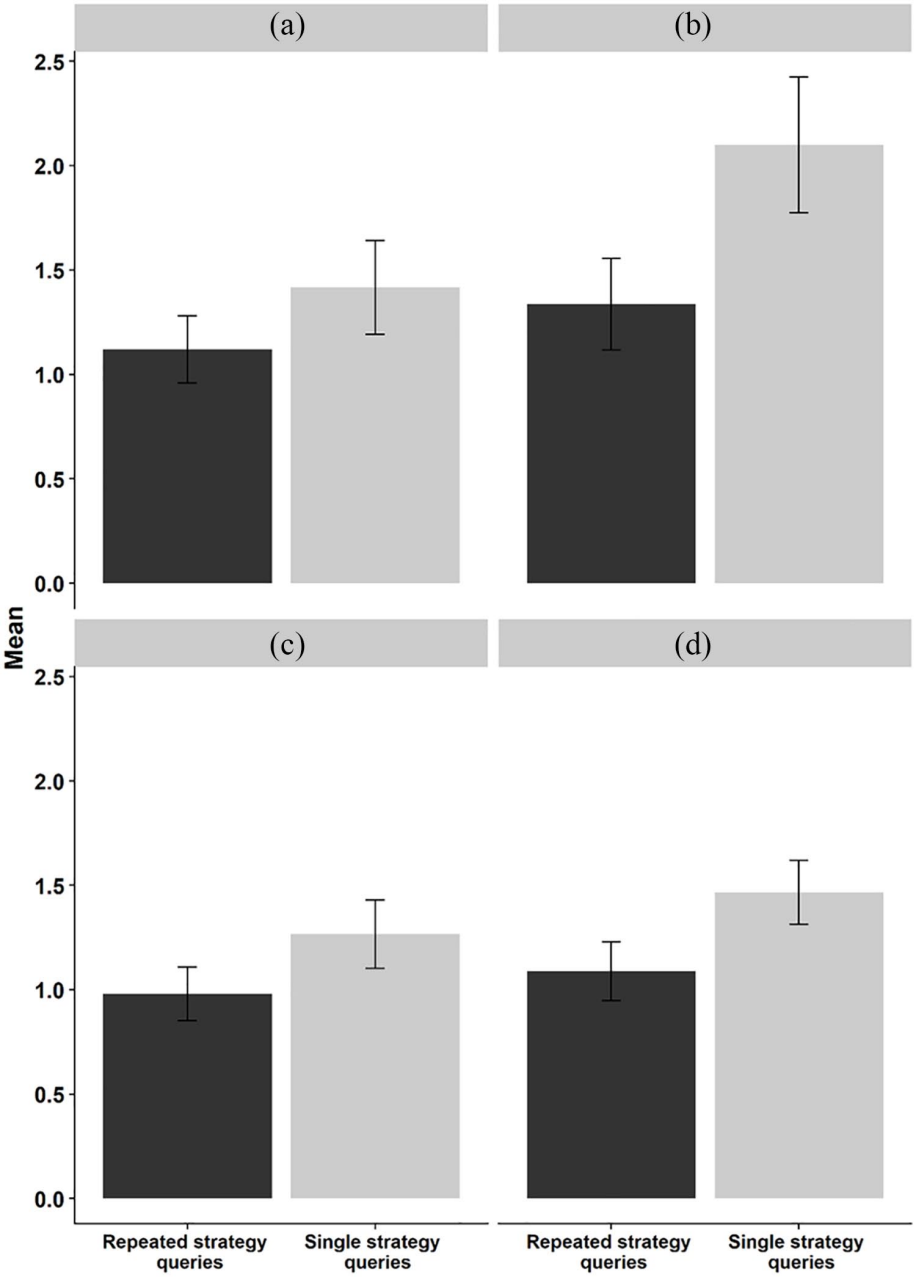
Strategy level of detail in (a) the pseudoword and the (b) real word condition as a function of group. Strategy sophistication in (c) the pseudoword and (d) the real word condition as a function group. Error bars represent 95% confidence intervals.

To examine whether repeated strategy reporting affected strategy use at the end of the task, we ran Bayesian ANOVA analyses with strategy use at the fifth block as the dependent variable and group as the independent variable. With respect to strategy LoD in the fifth block of the pseudoword task (see Figure 4(a)), the results showed weak evidence for a main effect of group (*M*_diff_ = 0.28, 95% HDI = [0.03, 0.55], BF_10_ = 1.22), whereas in the real word condition (see Figure 4(b)), the Bayes factor showed very strong evidence for a main effect of group (*M*_diff_ = 0.74, 95% HDI = [0.36, 1.10], BF_10_ > 150), with the SSQ group reporting higher LoD scores in the last block compared with the RSQ group. We also probed for possible group differences with strategy sophistication as a continuous dependent variable (see Figure 4(c) and (d)), where No strategy was coded as 0, Maintenance or other strategies as 1, and Manipulation as 2 (see Fellman et al., 2020). The results showed positive evidence for a group effect in the pseudoword condition (*M*_diff_ = 0.28, 95% HDI = [0.08, 0.48], BF_10_ = 4.25), and strong evidence for a group effect in the real word condition (*M*_diff_ = 0.36, 95% HDI = [0.16, 0.57], BF_10_ = 33.08), indicating that the SSQ group reported more sophisticated strategies in the fifth block compared with the RSQ group.

All in all, when compared with controls, repeated open-ended strategy queries were not reflected in objective recall performance. However, somewhat unexpectedly, the analyses of the two strategy variables revealed evidence for group differences so that those who repeatedly filled in the open-ended strategy query had lower levels of strategy sophistication and level of detail on the fifth block in both task conditions.

**Table 6.** Summary of the results in light of the two hypotheses and the replication attempt.

Prediction	Outcome	Description
*Hypothesis 1 (novelty hypothesis)*		
1.1. The pseudoword but not the word condition shows increase in the frequency of strategy use across the task blocks	Contradicted	Looking at the analysis that targeted the first two blocks, strategy use increased in both conditions.
1.2. The pseudoword but not the real word condition shows increase in LoD across the task blocks	Contradicted	No support for LoD increase in the two conditions either across all blocks or between the first two blocks.
1.3. Initial performance in the pseudoword condition: lower frequency of strategy use than in the real word condition	Supported*	In the pseudoword condition, 72.3% of participants reported using some strategy in the first block, whereas 85.2% did so in the real word condition.
1.4. Initial performance in the pseudoword condition: lower level of strategy detail than in the real word condition	Supported*	On average, the LoD score was initially approximately 0.5 points lower in the pseudoword condition than in the real word condition.
1.5. Initial performance in the pseudoword condition: lower recall than in the real word condition	Supported*	In the first block, participants recalled on average 7.8/18 real words and 2.5/10 pseudowords.
*Hypothesis 2 (task demand hypothesis)*		
2.1. Both task conditions: increase in the frequency of strategy use across the task blocks	Supported	Looking at the analysis that targeted the first two blocks, strategy use increased in both conditions.
2.2. Both task conditions: increase in level of strategy detail across the task blocks	Inconclusive	No support for LoD increase in the two conditions either across all blocks or between the first two blocks.
*Predictions shared by the two hypotheses*		
3.1. Recall performance across task blocks correlates positively with strategy use	Partly supported	In the real word condition, strategy use was associated with higher recall performance, but in the pseudoword condition the evidence was only weak.
3.2. Recall performance across task blocks correlates positively with LoD	Supported	In both conditions, higher LoD scores were associated with better recall performance.
*Replication (concerns only the pseudoword condition)*		
4.1. Strategies are actively used very early in a novel task.	Supported*	72.3% of participants reported using some strategy in the first block of the pseudoword learning task.
4.2. Strategy use increases and becomes more stable across blocks.	Supported	The analysis on the first two blocks showed an increase in strategy use. As regards stability, the number of strategy changers decreased as the task progressed (Figure 1(c)).
4.3. Strategy type is associated with task performance.	Inconclusive	Only weak evidence of a main effect of strategy so that strategy users (Manipulation and Maintenance/Other) performed better than No strategy users.
4.4. Strategy level of detail is associated with task performance	Supported	Higher LoD was associated with better recall performance.
4.5 Inconsistencies in agreement between open-ended and list-based strategy reports.	Supported*	The agreement was 55.3% for the pseudoword task in the present study, and 52.3% in the earlier study employing a working memory task

Supported/Contradicted: BF_10/01_ > 3; Supported*: based on descriptive evidence; Inconclusive: BF_10/01_ < 3; LoD: level of strategy detail.

## Discussion

In the present pre-registered study, we investigated the temporal pattern of strategy use and its role in recall performance in two episodic memory tasks that varied in stimulus novelty. Our study had three main aims: (1) to test two partly competing hypotheses (novelty hypothesis vs task demand hypothesis) regarding strategy development on a memory task, (2) to replicate our previous results regarding strategy use in a novel memory task, and (3) to examine whether repeated open-ended strategy queries affect task performance and/or strategy use. In what follows, we discuss the results separately for each of the main aims, followed by a consideration of study limitations and final conclusions.

### Triggers of strategy development

The first aim was to test whether strategy development in a memory task is triggered by task novelty as postulated by the cognitive routine framework (Gathercole et al., 2019; novelty hypothesis), or whether it suffices to have a demanding enough familiar task to elicit strategy development (task demand hypothesis). Gathercole and colleagues’ cognitive routine framework states that for new tasks, individuals must develop a new cognitive routine (i.e., strategy) following the principles of cognitive skill learning, whereas for familiar tasks, no new routine or strategy will be needed. In contrast, Hypothesis 2 states that novelty is not a sufficient criterion for determining whether a given task elicits strategy development. Besides novelty, one must take into account the demands set by the task. In other words, the task demand hypothesis supposes that a familiar task can also trigger strategy development when it is demanding enough.

To test these two hypotheses, we selected two tasks that differed only in stimulus novelty. While it is likely that an important contributor to novelty is the unfamiliarity of the task paradigm itself (cf. Gathercole et al., 2019), we sought to minimise the difference between the two task conditions to aid the interpretation of the results. Thus, we assumed that while learning a list of real words corresponds more to everyday activities familiar to most people, learning a list of meaningless pseudowords can be considered as a more novel task.

There were two predictions that were shared by both hypotheses: strategy type and strategy level of detail are related to objective recall performance. These predictions were supported, except for the relationship between strategy type and recall performance in the pseudoword condition. However, even for this prediction, the block-to-block average scores in the pseudoword condition appeared to be in line with the expectation, although there was high individual variance in performance within each strategy category (see Figure 3(a)). These results broadly concur with earlier research on the important role of strategies in episodic memory performance (e.g., Camp et al., 1983; Hill et al., 1990; McDaniel & Kearney, 1984), and motivates further research into the temporal pattern of strategy use within a task.

Concerning the competing predictions made by the two hypotheses, the results showed partial support for Hypothesis 2 (task demand hypothesis). In our moderately demanding word learning task, the frequency of strategy use showed an increase that was observed between the first two task blocks. This suggests that the familiar task was not performed with existing routines right from the start, but that the participants made an initial adjustment to their strategies to manage the task. Such a change was predicted by the task demand hypothesis, but not by the novelty hypothesis. This concurs with the idea that strategy behavior is elicited by cognitive tasks that participants perceive as moderately difficult (Belmont & Mitchell, 1987). Theory-wise, the introduction of task difficulty as another factor that (besides task novelty) can elicit strategy development can be seen as a complement to the cognitive routine framework by Gathercole et al. (2019). Note that frequency of strategy use in the familiar task diminished towards the end of the task: a likely reason for this is that the task was getting easier, with only a few additional words left to recall from the repeatedly presented list of common nouns.

The present evidence on the role of task difficulty in strategy development is only partial, as the other strategy measure, strategy level of detail, did not evidence a change across the blocks in either task condition. One possible reason for this is that strategy level of detail appears to be a less straightforward strategy measure than strategy use/non-use. That is, even though strategy level of detail is valid in the sense that it is a robust predictor of objective memory performance (Laine et al., 2018; Waris et al., 2021), it reflects strategy use in a more indirect way. For example, a less advanced strategy would earn high points in level of detail (but not as strategy type) if it is described thoroughly. Another possible reason is that participants who gave block-by-block strategy reports might have refrained from producing fully detailed reports if strategy-related refinements did not feel substantial enough. This line of reasoning seems to be supported by the pairwise comparison of the Repeated and Single Strategy Queries groups, where the latter group that was prompted with a strategy query only once reported using more sophisticated strategies and had higher level of detail scores in the strategy reports in the last block of each task, even though no task performance differences were observed. Hence, this could suggest that repeated open-ended reports might not capture all aspects of changes in strategy use.

### Replicating earlier findings on strategy use in a novel memory task

The attempt to replicate the results of Waris et al. (2021) with the present novel memory task (pseudoword list learning) was for the most part successful, as four of the five predictions gained support: (1) task-initial strategy use was evident right at the beginning of the novel task, (2) the proportion of strategy users increased in the first task blocks, (3) strategy level of detail was associated with novel task performance, and (4) there was some inconsistency between open-ended versus list-based strategy reports.

With respect to point 1, it is worth underscoring that increase in strategy use was evident only for the first two blocks, but the overall pattern was similar to Waris et al. (2021). It is also important to point out that the memory tasks as well as the analytical approach in these two studies were quite different. Waris et al. used an adaptive working memory updating task (n-back) and a Null Hypothesis Significant Testing approach, while this study employed a non-adaptive episodic memory (list learning) task together with a Bayesian Inference (BF) approach. Given the fact that the same findings were replicated using a Bayesian approach (which generally requires more robust empirical evidence for observing an effect if present; see, for example, Wagenmakers et al., 2018), and even extended it to the present familiar word learning task that was outside the scope of the replication attempt, these findings appear to represent more general features of strategy deployment in memory tasks. The present results suggest that the Formation stage (Chein & Schneider, 2012) where the metacognitive system selects the way the task is managed (i.e., the strategy) is very short-lived for typical memory tasks, taking place within the first minutes into the task.

### Effects of repeated open-ended strategy responses on task performance

Our third main aim was to test whether repeated open-ended strategy queries affect task performance and/or strategy use. Waris et al. (2021) found that repeated list-based strategy reports were associated with better task performance, but they did not have a control condition for the repeated open-ended strategy reports. As compared with controls who were not giving open-ended strategy reports throughout the task, this study found no difference in objective recall. For further research, the aggregated evidence from this study and the one by Waris et al. (2021) suggest that open-ended strategy reports do not bias memory performance, which makes them more recommendable for repeated testing than list-based reports. However, the participants who had repeatedly filled out the open-ended strategy reports showed lower values of strategy sophistication and level of detail on the fifth block in both task conditions, which possibly reflects a certain degree of saturation and fatigue for completing the open-ended strategy report for the fifth time (i.e., these participants possibly refrained from reporting perceived smaller strategy-related changes and/or showed some weariness that resulted in fewer reports).

### Study limitations

One limitation of this study is the way task novelty versus familiarity was defined. It is possible that our results would have been more clearcut had we chosen to contrast two different memory task paradigms that would vary in novelty, rather than keeping the paradigm constant and varying the novelty of the stimuli. However, different task paradigms can call for quite different cognitive processes and strategic demands, making it more difficult to compare them directly to each other. Moreover, one could ask how familiar the specific real word learning task actually was to the participants. We did probe this with a question “Before this study, have you ever completed a comparable word list task with **real words**?” Only 19.8% of the participants replied positively to this question, but it is not clear that they would have considered related list learning variants (shopping lists, to-do-lists, name lists, etc.) from their everyday life when responding to the question. As regards the pseudoword task, the corresponding percentage was zero. Be it as it may, given how Gathercole and colleagues define novelty in a memory task (“requiring participants to store material in highly unfamiliar and challenging cognitive conditions,” p. 24), it appears that acquisition of a list of common nouns cannot be taken as a novel task. Further studies that systematically vary the novelty and difficulty of both task paradigm and stimuli will shed more light on the role of these factors in strategic behavior.

A potential factor affecting strategy use, strategy reporting, and task performance is motivation. Highly motivated individuals could put time into describing their strategy and they could also put effort into performing optimally. In this study, participants were asked to report their motivation for completing the learning tasks after completing both tasks. We conducted post hoc analyses to study this issue (see Supplementary material). These analyses provided weak-to-positive evidence against a main effect of motivation on task performance in both task conditions. However, in the real word condition, motivation interacted with block (across all five blocks BF_10_ = 6.96; across the two first blocks BF_10_ = 6.95), indicating that the more motivated participants tended to gain more across blocks as compared with the less motivated participants. Moreover, for both task conditions, evidence for an effect of motivation on our strategy measures were either weak or supported the H_01_ with weak or positive evidence. Thus, evidence for motivational effects was very limited in this study. However, this does not eliminate the fact that motivation represents one important background factor that can act as a catalyst in cognitive performance.

### Final conclusion

To sum up, the present findings add to the earlier literature on the relevance of memory strategies in episodic memory performance (e.g., Camp et al., 1983; Hill et al., 1990; McDaniel & Kearney, 1984). More importantly, together with our earlier block-by-block strategy analysis of an adaptive memory updating task (Waris et al., 2021), they paint a picture of a very early dynamic phase in strategy employment, taking place within the first few minutes into a memory task that can be either a novel or more or less familiar but difficult enough. Strategies are adopted and changed particularly during this short-lived initial phase, albeit task demands keep changing throughout the task with gradually increasing performance (the present episodic memory task getting easier as the same list is presented repeatedly; the adaptive memory updating task used by Waris et al., 2021, getting more difficult). This fits well to the general skill learning view that identifies the first Formation phase where strategies are established by the metacognitive system (Chein & Schneider, 2012; see also Taatgen, 2013). It seems logical that in memory tasks that do not require problem-solving, this phase is short-lasting. Strategic decisions made at the Formation phase have important consequences, as strategy choices are related to the objective outcomes of a memory task. At a more general level, the present results speak for a more dynamic view on cognition (Beer, 2000; Riley & Holden, 2012; Smith & Thelen, 2003; Van Gelder, 1998) and the utility in analysing test performances at a more detailed temporal scale to understand how we adapt to task demands and how the final performance outcome is shaped.

## Supplemental Material

sj-docx-1-qjp-10.1177_1747021820980301 – Supplemental material for Stimulus novelty, task demands, and strategy use in episodic memory Click here for additional data file. Supplemental material, sj-docx-1-qjp-10.1177_1747021820980301 for Stimulus novelty, task demands, and strategy use in episodic memory by Otto Waris, Daniel Fellman, Jussi Jylkkä and Matti Laine in Quarterly Journal of Experimental Psychology

## Notes

1. Big Five Openness and Conscientiousness items from the BFI-2 (Soto & John, 2017), International Personality Item Pool (Goldberg, 1999; Goldberg et al., 2006) representations of the Values in Action Originality/Creativity scale (Peterson & Seligman, 2004) and Cloninger’s Temperament and Character Inventory Dependence and Competence scales (Cloninger et al., 1994).

2. For the picture description task, we first scanned the descriptions for four keywords, and two independent raters checked those responses that included one or none of the keywords (*n* = 18). Both raters were in agreement that two of the responses were irrelevant/lacking any actual detail.

3. We decided to depart from our pre-registered stricter criteria (i.e., no deviation), as it would have resulted in the exclusion of 19 additional participants, and somewhat inaccurate/inconsistent characterisations of one’s education are possible, as educational backgrounds and their comparability vary widely.

4. These corrupted cases were in the Single strategy queries group, where some participants wrote their strategy reports in wrong response boxes, and the raters could not tell which description referred to which task.
