# Supplementary material for: Stimulus novelty, task demands, and strategy use in episodic memory
Source: Q J Exp Psychol (Hove). 2021 Jan 9;74(5):872–88. doi: 10.1177/1747021820980301 (PMC8054168; doi:10.1177/1747021820980301)
Supplement: sj-docx-1-qjp-10.1177_1747021820980301 – Supplemental material for Stimulus novelty, task demands, and strategy use in episodic memory [file sj-docx-1-qjp-10.1177_1747021820980301.docx]

**Supplementary Material**

Waris, Fellman, Jylkkä, & Laine. Stimulus novelty, task demands, and strategy use in episodic memory

**Posttest Strategy Questionnaire: Real words**

Which following option(s) best describes your way of memorizing the words in the **last (fifth)** list of the word list task with **real words?** That is, did you do something to help you memorize the words? Please mark **all the methods** that you used by ticking the appropriate box(es).

**Rehearsal / Repetition**: I actively repeated the words out loud or in my mind.

**Grouping**: I grouped words into larger units.

**Visualization**: I mentally 'saw' images of what the words represent.

**Spatial association**: I 'placed' each word in a familiar location (for example, on the street I live).

**Semantic association**: I grouped together words that, for me, share a meaningful connection. An example would be to think of furniture to help recall the words chair, table, and shelf.

**Narrative**: I used the words to create a story.

**Instinct**: I did not try to actively memorize the words, but rather 'went with the flow' and noted each word as it appeared.

**Selective focus**: I focused on remembering only a subset of the words and disregarded the rest.

**Guessing**: I just entered some random words.

**Other strategy**: I used a strategy that is not listed here.

**No strategy**: I had no strategy.

**Did not understand**: I did not understand what I was supposed to do.

-------------------------------------------------------------------------------------------------

In the **last (fifth)** list of the word list task with **real words,** what method did you use **most often?** You can select only one option.

**Rehearsal / Repetition**: I actively repeated the words out loud or in my mind.

**Grouping**: I grouped words into larger units.

**Visualization**: I mentally 'saw' images of what the words represent.

**Spatial association**: I 'placed' each word in a familiar location (for example, on the street I live).

**Semantic association**: I grouped together words that, for me, share a meaningful connection. An example would be to think of furniture to help recall the words chair, table, and shelf.

**Narrative**: I used the words to create a story.

**Instinct**: I did not try to actively memorize the words, but rather 'went with the flow' and noted each word as it appeared.

**Selective focus**: I focused on remembering only a subset of the words and disregarded the rest.

**Guessing**: I just entered some random words.

**Other strategy**: I used a strategy that is not listed here.

**No strategy**: I had no strategy.

**Did not understand**: I did not understand what I was supposed to do.

-------------------------------------------------------------------------------------------------

In the **last (fifth)** list of the word list task with **real words,** what was the **most sophisticated** method you used? You can select only one option.

**Rehearsal / Repetition**: I actively repeated the words out loud or in my mind.

**Grouping**: I grouped words into larger units.

**Visualization**: I mentally 'saw' images of what the words represent.

**Spatial association**: I 'placed' each word in a familiar location (for example, on the street I live).

**Semantic association**: I grouped together words that, for me, share a meaningful connection. An example would be to think of furniture to help recall the words chair, table, and shelf.

**Narrative**: I used the words to create a story.

**Instinct**: I did not try to actively memorize the words, but rather 'went with the flow' and noted each word as it appeared.

**Selective focus**: I focused on remembering only a subset of the words and disregarded the rest.

**Guessing**: I just entered some random words.

**Other strategy**: I used a strategy that is not listed here.

**No strategy**: I had no strategy.

**Did not understand**: I did not understand what I was supposed to do.

-------------------------------------------------------------------------------------------------

**Posttest Strategy Questionnaire: Pseudowords**

Which following option(s) best describes your way of memorizing the words in the **last (fifth)** list of the word list task with **pseudowords** (the made-up words)? That is, did you do something to help you memorize the words? Please mark **all the methods** that you used by ticking the appropriate box(es).

**Rehearsal / Repetition**: I actively repeated the words out loud or in my mind.

**Grouping**: I grouped words into larger units.

**Visualization**: I mentally ”saw” images of what I thought the words could represent.

**Spatial association**: I 'placed' each word in a familiar location (for example, on the street I live).

**Verbal association**: I tried to associate or link the made up words with real words.

**Narrative**: I used the words to create a story, even though the words were made up.

**Instinct**: I did not try to actively memorize the words, but rather 'went with the flow' and noted each word as it appeared.

**Selective focus**: I focused on remembering only a subset of the words and disregarded the rest.

**Guessing**: I just entered some random words.

**Other strategy**: I used a strategy that is not listed here.

**No strategy**: I had no strategy.

**Did not understand**: I did not understand what I was supposed to do.

-------------------------------------------------------------------------------------------------

In the **last (fifth)** list of the word list task with **pseudowords** (the made-up words), what method did you use **most often?** You can select only one option.

**Rehearsal / Repetition**: I actively repeated the words out loud or in my mind.

**Grouping**: I grouped words into larger units.

**Visualization**: I mentally ”saw” images of what I thought the words could represent.

**Spatial association**: I 'placed' each word in a familiar location (for example, on the street I live).

**Verbal association**: I tried to associate or link the made up words with real words.

**Narrative**: I used the words to create a story, even though the words were made up.

**Instinct**: I did not try to actively memorize the words, but rather 'went with the flow' and noted each word as it appeared.

**Selective focus**: I focused on remembering only a subset of the words and disregarded the rest.

**Guessing**: I just entered some random words.

**Other strategy**: I used a strategy that is not listed here.

**No strategy**: I had no strategy.

**Did not understand**: I did not understand what I was supposed to do.

-------------------------------------------------------------------------------------------------

In the **last (fifth)** list of the word list task with **pseudowords** (the made-up words), what was the **most sophisticated** method you used? You can select only one option.

**Rehearsal / Repetition**: I actively repeated the words out loud or in my mind.

**Grouping**: I grouped words into larger units.

**Visualization**: I mentally ”saw” images of what I thought the words could represent.

**Spatial association**: I 'placed' each word in a familiar location (for example, on the street I live).

**Verbal association**: I tried to associate or link the made up words with real words.

**Narrative**: I used the words to create a story, even though the words were made up.

**Instinct**: I did not try to actively memorize the words, but rather 'went with the flow' and noted each word as it appeared.

**Selective focus**: I focused on remembering only a subset of the words and disregarded the rest.

**Guessing**: I just entered some random words.

**Other strategy**: I used a strategy that is not listed here.

**No strategy**: I had no strategy.

**Did not understand**: I did not understand what I was supposed to do.

-------------------------------------------------------------------------------------------------

**Supplementary analyses regarding motivation, strategy use and task performances**

For elucidating whether motivation was related to increases in strategy use and actual memory performance across time, we employed LME models. In the present study, motivation was probed at the end of the study by asking each participant “On a scale from 1 (not at all) to 10 (very), how motivated were you to perform the word list tasks?”. Hence, motivation was not probed separately for each task.

**Motivation analyses across all five task blocks**

*LoD predicted by motivation*

First, we let LoD be regressed on motivation and block, together with their interaction term. In the pseudoword condition, we found weak evidence for a main effect of motivation on LoD (M_diff_ = 0.05 95 % HDI [-0.03 – 0.12] BF_10_ = 1.04 ± 1.77%) and positive evidence against a Motivation × Block interaction (M_diff_ = -0.01 95 % HDI [-0.04 – 0.03] BF_01_ = 5.56 ± 2.59%). The results were broadly similar in the real word condition: we found weak evidence against a main effect of motivation on LoD (M_diff_ = 0.04 95 % HDI [-0.06 – 0.13] BF_01_ = 1.64 ± 2.01%), and positive evidence against a Motivation × Block interaction (M_diff_ = 0.00 95 % HDI [-0.05 – 0.04] BF_01_ = 5.56 ± 1.43%).

*Proportional strategy increase predicted by motivation*

We also probed whether the proportional strategy increase across blocks was related to motivation. For the pseudoword condition, we found weak evidence for a main effect of motivation on strategy proportion (M_diff_ = 0.03 95 % HDI [-0.01 – 0.07] BF_10_ = 1.34 ± 9.08%), and weak evidence against a Motivation × Block interaction (M_diff_ = -0.01 95 % HDI [-0.03 – 0.01] BF_01_ = 2.50 ± 3.50%). For the real word condition, we observed weak evidence against a main effect of motivation on strategy proportion (M_diff_ = 0.02 95 % HDI [-0.01 – 0.05] BF_01_ = 1.39 ± 4.89%), and positive evidence against a Motivation × Block interaction (M_diff_ = 0.00 95 % HDI [-0.01 – 0.02] BF_01_ = 5.00 ± 2.02%).

*Memory performance predicted by motivation*

Lastly, we examined whether motivation was related to actual memory performance, with the recall score serving as dependent variable, and block and motivation and their interaction as predictors. For the pseudoword condition, we observed weak evidence against a main effect of motivation on recall performance(M_diff_ = 0.17 95 % HDI [-0.05– 0.40] BF_01_ = 2.38 ± 0.96%), and positive evidence against a Motivation × Block interaction (M_diff_ = 0.08 95 % HDI [-0.02 – 0.17] BF_01_ = 4.55 ± 0.88%). For the real word condition, we found positive evidence against a main effect of motivation (M_diff_ = 0.20 95 % HDI [-0.11 – 0.54] BF_01_ = 3.12 ± 1.21%), but positive evidence for a Motivation × Block interaction (M_diff_ = 0.25 95 % HDI [0.10 – 0.41] BF_10_ = 6.96 ± 1.21%). This indicates that the more motivated participants showed larger performance increases in the real word condition across blocks as compared to the less motivated participants.

**Motivation analyses across the first two task blocks**

*LoD predicted by motivation*

In the pseudoword condition, we found weak evidence for a main effect of motivation on LoD (M_diff_ = 0.05 95 % HDI [-0.02 – 0.12] BF_10_ = 1.05 ± 1.56%) and positive evidence against a Motivation × Block interaction (M_diff_ = -0.01 95 % HDI [-0.04 – 0.03] BF_01_ = 5.26 ± 5.54%). The results were largely the same in the real word condition: we found weak evidence against a main effect of motivation on LoD (M_diff_ = 0.04 95 % HDI [-0.06 – 0.13] BF_01_ = 1.56 ± .53%), and positive evidence against a Motivation × Block interaction (M_diff_ = 0.00 95 % HDI [-0.05 – 0.05] BF_01_ = 5.56 ± 0.62%).

*Proportional strategy increase predicted by motivation*

For the pseudoword condition, we found weak evidence for a main effect of motivation on strategy proportion (M_diff_ = 0.03 95 % HDI [-0.01 – 0.07] BF_10_ = 1.40 ± 1.13 %), and positive evidence against a Motivation × Block interaction (M_diff_ = -0.01 95 % HDI [-0.03 – 0.01] BF_01_ = 2.38 ± 2.05%). For the real word condition, we observed weak evidence against a main effect of motivation on strategy proportion (M_diff_ = 0.02 95 % HDI [-0.01 – 0.05] BF_01_ = 1.32 ± 1.40%), and positive evidence against a Motivation × Block interaction (M_diff_ = 0.00 95 % HDI [-0.01 – 0.02] BF_01_ = 5.00 ± 1.36%).

*Memory performance predicted by motivation*

For the pseudoword condition, we observed weak evidence against a main effect of motivation on recall performance (M_diff_ = 0.17 95 % HDI [-0.05 – 0.41] BF_01_ = 2.38 ± 0.88%), and positive evidence against a Motivation × Block interaction (M_diff_ = 0.08 95 % HDI [-0.01 – 0.18] BF_01_ = 4.55 ± 0.94%). For the real word condition, we found positive evidence against a main effect of motivation (M_diff_ = 0.21 95 % HDI [-0.14 – 0.53] BF_01_ = 3.12 ± 1.17%), but positive evidence for a Motivation × Block interaction (M_diff_ = 0.25 95 % HDI [0.09 – 0.041] BF_10_ = 6.95 ± 1.09%). This indicates that the more motivated participants showed larger performance increases from the first to second block in the real word condition as compared to the less motivated participants.
